# HSBP1 Is a Novel Interactor of FIP200 and ATG13 That Promotes Autophagy Initiation and Picornavirus Replication

**DOI:** 10.3389/fcimb.2021.745640

**Published:** 2021-11-15

**Authors:** Mario Mauthe, Nilima Dinesh Kumar, Pauline Verlhac, Nicole van de Beek, Fulvio Reggiori

**Affiliations:** ^1^ Department of Biomedical Sciences of Cells & Systems, Molecular Cell Biology Section, University Medical Center Groningen, University of Groningen, Groningen, Netherlands; ^2^ Department of Medical Microbiology and Infection Prevention, University Medical Center Groningen, University of Groningen, Groningen, Netherlands

**Keywords:** autophagy, infection, ULK kinase complex, EMCV, CVB3, EV71

## Abstract

ATG13 and FIP200 are two subunits of the ULK kinase complex, a key regulatory component of the autophagy machinery. We have previously found that the FIP200-ATG13 subcomplex controls picornavirus replication outside its role in the ULK kinase complex and autophagy. Here, we characterized HSBP1, a very small cytoplasmic coiled-coil protein, as a novel interactor of FIP200 and ATG13 that binds these two proteins *via* FIP200. HSBP1 is a novel pro-picornaviral host factor since its knockdown or knockout, inhibits the replication of various picornaviruses. The anti-picornaviral function of the FIP200-ATG13 subcomplex was abolished when HSBP1 was depleted, inferring that this subcomplex negatively regulates HSBP1’s pro-picornaviral function during infections. HSBP1depletion also reduces the stability of ULK kinase complex subunits, resulting in an impairment in autophagy induction. Altogether, our data show that HSBP1 interaction with FIP200-ATG13-containing complexes is involved in the regulation of different cellular pathways.

## Brief Research Report

## Introduction

Macroautophagy (hereafter referred to as autophagy) is a cellular degradation pathway that is evolutionary conserved (Lahiri et al., 2019). This process is characterized by the selective or non-selective sequestration of cytoplasmic cargoes within double-membrane autophagosomes, which subsequently fuse with lysosomes to deliver their cargo into the hydrolytic interior of these organelles (Dikic and Elazar, 2018; Nakatogawa, 2020). Autophagy is active at basal levels in every eukaryotic cell and can be enhanced by stresses such as nutrient starvation and pathogen infection (Galluzzi et al., 2014; Deretic, 2021). Autophagosome biogenesis is orchestrated by the autophagy-related (ATG) proteins (Nakatogawa, 2020). Four of them, the kinase unc-51 like autophagy activating kinase (ULK) 1 (or ULK2), ATG13, RB1 inducible coiled-coil 1 (FIP200/RB1CC1) and ATG101, form the ULK kinase complex, which is a key regulator of autophagy induction (Licheva et al., 2021). Activation of the ULK kinase complex initiates a signaling cascade that leads to the formation of autophagosomes (Licheva et al., 2021).

Numerous ATG proteins have other functions than the ones in autophagy (Bestebroer et al., 2013; Mauthe and Reggiori, 2016; Galluzzi and Green, 2019). In a previous study, we performed a siRNA-based screen to identify in an unbiased fashion the extent of the non-autophagic roles of the ATG proteins. In particular, we examined the impact of the depletion of each component of the ATG proteome on the replication of 6 viruses from 6 different virus families. With this approach, we also identified an anti-viral role of the subcomplex formed by ATG13 and FIP200, outside the context of the ULK kinase complex (Mauthe et al., 2016). This anti-viral role is specific for picornaviruses, a large virus family of non-enveloped, small (~30 nm in diameter) viruses with a positive-stranded RNA genome, which cause diseases in humans and animals (Mauthe et al., 2016). Picornaviruses are classified into different genera based on a complex set of rules (ICTV taxonomy) (Zell et al., 2017). Examples of genera are enteroviruses, which include coxsackievirus B3 (CVB3) and enterovirus 71 (EV71), and cardioviruses, a member of which is the encephalomyocarditis virus (EMCV) (Zell et al., 2017).

In this study, we have investigated heat shock factor binding protein 1 (HSBP1), a protein that we found binding to FIP200 and ATG13 (Mauthe et al., 2016), to gain additional insights into the anti-picornaviral function of the FIP200-ATG13 complex. HSBP1 is a small coiled-coil protein of 12 kDa that forms trimers (Liu et al., 2009). HSBP1 has been identified as a negative regulator of heat shock factor 1 (HSF1) (Satyal et al., 1998), the primary mediator of transcriptional responses to proteotoxic stresses (Vihervaara and Sistonen, 2014). Recently, it has also been shown that HSBP1 is crucial for the assembly of the Wiskott–Aldrich syndrome protein and SCAR homolog (WASH) complex (Visweshwaran et al., 2018), an actin-regulating complex that is recruited to endosomes by interaction with the retromer complex, that plays a role in endosomal protein sorting (Seaman et al., 2013). Here, we identified two new functions of HSBP1. First, we found that HSBP1 is important for the stability of ULK kinase complex subunits and therefore for autophagy initiation as well. Second, HSBP1 is a pro-picornaviral host factor that dissociates from the cytoplasmic FIP200-ATG13 subcomplex and translocates into the nucleus after picornavirus infection. We also provide evidence that the pro-picornaviral role of HSBP1 could be negatively controlled by the FIP200-ATG13 subcomplex, which thereby acts as an anti-picornaviral factor.

## Material and Methods

### Antibodies and Reagents

The following primary antibodies were used: rabbit anti-LC3 (Novus Biologicals, Littleton, CO), mouse anti-LC3 (Nanotools, Teningen, Germany), mouse anti-p62 (Abcam, Cambridge, UK), mouse anti-tubulin (Sigma-Aldrich, St. Louis, MO), rabbit anti-ATG13 (Sigma-Aldrich), mouse anti-HSBP1 (Sigma-Aldrich, clone 2C3), rabbit anti-ATG16L1 (MBL, Woburn, MA), mouse anti-actin (Merck, Darmstadt, Germany), mouse anti-GFP (Clontech, Shiga, Japan), rabbit anti-ULK1 (Santa Cruz, Dallas, TX), rabbit anti-FIP200 (Bethyl Laboratories, Montgomery, TX), mouse anti-enterovirus (DAKO, Glostrup, Denmark), mouse anti-dsRNA (English & Scientific Consulting Bt., Budapest, Hungary), rabbit anti-capsid (EMCV, a kind gift from Ann Palmenberg, University of Wisconsin, Madison, WI), mouse anti-VP1 (EMCV, a kind gift from Hanchun Yang, China Agricultural University, Beijing, China), mouse anti-4G2 (Dengue virus (DENV) and Zika virus (ZIKV), Merk Millipore, Billerica, MA) and rabbit anti-E1 (Chikungunya virus (CHIKV), from Jolanda Smit, University Medical Center Groningen, The Netherlands), mouse anti-NP (influenza A virus (IAV), BioRad, Hercules, CA). The secondary antibodies were from Thermo Fisher Scientific (Waltham, MA) and were AlexaFluor488-conjugated goat anti-mouse or chicken anti-rabbit; AlexaFluor568-conjugated goat anti-mouse or donkey anti-rabbit; AlexaFluor680-conjugated goat anti-mouse or goat anti-rabbit; and AlexaFluor800-conjugated goat anti-mouse secondary antibodies were used for the visualization of the primary antibodies, and they were all from Thermo Fisher Scientific (Waltham, MA). Hoechst33342 was from Sigma Aldrich while bafilomycin A_1_ (BafA1) was from BioAustralis (Smithfield NSW, Australia).

To induce autophagy, cells were washed two times with Earle’s balanced salt solution (EBSS, Sigma-Aldrich) and then incubated in the same medium for 2 h.

### Virus Stocks and Infection

Virus stocks of EMCV, EMCV-Zn, Rluc-EMCV, CVB3, RLuc-CVB3, EV71 (kind gifts from Frank van Kuppeveld, University of Utrecht, Netherlands), A/Puerto Rico/8/1934 (H1N1) IAV (kind gift from Anke Huckriede, University Medical Center Groningen, The Netherlands), DENV-2 strain 16681, ZIKV (clinical isolate from Surinam) and CHIKV (La Reunion OPY1) (all a kind gift from Jolanda Smit, University Medical Center Groningen, The Netherlands) were generated and propagated as described previously (Mauthe et al., 2016; Bhide et al., 2019; Diosa-Toro et al., 2019; Troost et al., 2020).

Virus infections for EMCV, EMCV-Zn wt, Rluc-EMCV, CVB3, RLuc-CVB3 were performed at a multiplicity of infection (moi) of 0.25 (or 1 for the co-immunoprecipitation experiments) and virus inoculums were left onto the cells for 6 h. For DENV, CHIKV, ZIKV and IAV, cells were infected at a moi of 0.8 for 26 h, at a moi of 10 for 10 h, at a moi of 0.8 for 26 h and at a moi of 0.5 for 6 h, respectively.

### Cloning

GFP-HSBP1 was generated by cloning HSBP1 cDNA (Source BioScience, Nottingham, UK, cat# IRQMp5018C039D) into the pEGFP-C1 vector as EcoRI/SacII fragment. Construct correctness was confirmed by DNA sequencing and protein expression by western blot (WB).

### Cell Lines and Cell Culture

U2OS (a kind gift from Ger Strous), HeLa (a kind gift from Peter van der Sluijs), U2OS cells stably expressing either GFP (GFP U2OS) or GFP-HSBP1 (GFP-HSBP1 U2OS), *hsbp1^-/-^* U2OS (HSBP1KO), *atg7^-/-^* U2OS (ATG7KO) (Janssen et al., 2018), *atg13^-/-^* U2OS (ATG13KO) cells and HeLa RFP-GFP-LC3 cells (a kind gift of Tamotsu Yoshimori) were cultured in Dulbecco’s Modified Eagle Medium (DMEM, Life Technology, Carlsbad, CA) supplemented with 100 U/ml penicillin, 100 μg/ml streptomycin and 10% fetal calf serum (FCS), at 37°C in 5% CO_2_ humidified atmosphere. The culture medium of HeLa RFP-GFP-LC3, GFP U2OS and GFP-HSBP1 U2OS was supplemented with 0.6 µg/ml G418 (Thermo Fisher Scientific). The HSBP1KO and ATG13KO cells were generated as previously described (Janssen et al., 2018). In brief, for the generation of HSBP1KO and ATG13KO cells using the CRISPR/Cas9 system, guides targeting exon 1 (CGTCCCTTACCACCGAGGTGAGG) and exon 2 (GGATATTTCTCCCAATGATCTGG) of HSBP1 and exon 3 (TTTGCTTCATGTGTAACCTCTGG and AGTCGGGAGGTCCATGTGTGTGG) of ATG13, respectively, were designed using optimized CRISPR design (http://crispr.mit.edu/). Guides were cloned into pX458 plasmid (Addgene #48138) allowing expression of guide RNAs and Cas9 along with GFP. U2OS cells were transfected for 48 h and subsequently clonally sorted based on GFP expression using a SH800S Cell Sorter (Sony biotechnology, San Jose, CA). Clones were then sequenced and protein expression was assessed by WB to verify the deletion of *HSBP1* and *ATG13*. Characterization of the ATG13KO cells can be found in **Figure S2C**.

To generate stable GFP or GFP-HSBP1 U2OS cells, U2OS cells were transfected with the pEGFP or the pEGFP-HSBP1 plasmid, respectively and then selected in a medium containing 0.6 µg/ml G418 for 10 days, resulting in a stable GFP U2OS or GFP-HSBP1 U2OS bulk population.

### Co-Immunoprecipitations

U2OS cells either stably or transiently expressing EGFP and EGFP-HSBP1 and grown in a 10 cm dish, were treated with EBSS for 2 h, EMCV infected for 6 h or left untreated before being subjected to lysis on ice in the following buffer: 20 mM Tris-HCl, pH 7.4, 150 mM NaCl, 2 mM MgCl_2_, 5 mM DTT, 0.5% Tween, Complete protease inhibitor (Roche, Basel, Switzerland), 1 mM PMSF. Co-immunoprecipitations were performed using the GFP‐trap beads (Chromotek, Planegg, Germany). Beads were incubated with the lysates for 2 h at 4°C and washed with the washing buffer (20 mM Tris-HCl, pH 7.4, 250 mM NaCl, 2 mM MgCl_2_, 5 mM DTT, 0.5% Tween-20). Proteins were eluted by boiling the beats in Laemmli loading buffer (65.8 mM Tris-HCl, pH 6.8, 26.3% glycerol, 2.1% SDS, 0.01% bromophenol blue) (Laemmli, 1970) and then examined by WB.

### Western-Blot Analyses

Cells grown in 6-well or 24-well plates were washed with PBS and harvested in 100 µl of lysis buffer (20 mM Tris-HCl, pH 7.6, 130 mM NaCl, 1% Triton-X100, Complete protease inhibitor). The lysates were incubated on ice for 30 min, vortexed and centrifuged at 14,000 g for 10 min at 4°C. Supernatants were finally collected and mixed with the Laemmli loading buffer. Alternatively, cells were directly lysed in the Laemmli loading buffer and sonicated for 1 min. Equal protein amounts were separated by SDS-PAGE and after WB, proteins were detected on PDVF membranes (Merck) using specific antibodies and the Odyssey Imaging System (LI-COR Biosciences, Lincoln, NE). Densitometric values of the bands were quantified on WB images at non-saturating exposures using the ImageJ software (Schneider et al., 2012), and normalized against the loading control. For the detection of the small HSBP1 protein, we followed an optimized protocol previously described (Visweshwaran et al., 2018). In brief, after running, the gels were put into a renaturation buffer (20% glycerol, 50 mM Tris–HCl, pH 7.4) for 30 min at room temperature and after transfer, PVDF membranes were incubated in PBS containing 0.4% paraformaldehyde for 30 min to cross-link the proteins before proceeding with the detection.

### Immunofluorescence Microscopy

Cells were fixed with 3.7% paraformaldehyde or 100% methanol, washed and blocked with blocking buffer (PBS, 1% bovine serum albumin, 0.1% saponin). Primary and secondary antibodies were diluted in the blocking buffer and incubated for 1 h at room temperature. Nuclei were stained with Hoechst33342 during the incubation with the secondary antibody for automated image acquisition. Alternatively, nuclei were stained with DAPI (Thermo Fisher Scientific) for confocal microscopy. Fluorescent microscopy images were collected with a DeltaVision RT fluorescence microscope (Applied Precision, Issaquah, WA) equipped with a CoolSNAP HQ camera (Photometrix, Kew, Australia). Images were generated by collecting a stack of 6 to 16 images with focal planes 0.30 μm apart, and subsequently deconvolved using the SoftWoRx software (Applied Precision). Quantification of puncta number was performed using the Icy software (http://icy.bioimageanalysis.org) using spot detector plugin or the ImageJ software. For automatic acquisition, fluorescence images were automatically acquired using a TissueFAXS (TissueGnostics, Vienna, Austria), which is based on a high-end fully motorized Zeiss AxioObserver Z1 microscope with a Zeiss- LD “Plan-Neofluar” 20x/0,4 Corr Dry objective (Zeiss, Oberkochen, Germany). The following filters were used: DAPI for the imaging of the nuclei, GFP for the acquisition of the GFP-HSBP1 and LC3 signals, and TexasRed for the imaging of the p62, EMCV and CVB3 capsid signal. The GFP and TexasRed filter were used for the GFP-RFP-LC3 tandem analysis. The acquired images were analyzed using the TissueQuest fluorescence analysis software (TissueGnostics GmbH, Vienna, Austria) to determine the cell count (based on the nuclei staining), the percentage of infected cells (based on the signal intensity in the cells), the mean signal intensity of infected cells and the translocation of GFP-HSBP1 into the nucleus (based on the signal intensities of GFP in the cytosol versus the nucleus). LC3 and p62 puncta were automatically quantified using the icy bioimage analysis software (De Chaumont et al., 2012).

### siRNA and DNA Transfections

U2OS cells were transfected for 48 h with 20 nM of either control siRNA or siRNA targeting HSBP1 (SMARTpool from Dharmacon, Lafayette, CO) using 0.1 µl, 0.5 µl or 2 µl of Lipofectamine RNAiMAX (Thermo Fisher Scientific) for 96-, 24- or 6-wells plate cultures, respectively, according to the manufacturer’s protocol. For the GFP and GFP-HSBP1 transfection, U2OS cells were seeded in 10 cm dishes (for co-immunoprecipitation, Co-IP) or in 6-well plates (for making stable cell lines), followed by a transfection procedure with Fugene (Promega, Madison, WI), according to the manufacturer’s protocol.

### Luciferase Assays

Cells grown in 96-well plates were washed with PBS and incubated with 50 µl of Lysis buffer (Thermo Fisher Scientific) at room temperature for 15 min, before storing the cell lysates at -20°C. 25 µl aliquots of thawed cell lysates were then used to measure renilla luciferase expression using the Renilla luciferase flash assay kit (Thermo Fisher Scientific). Alternatively, renilla luciferase activity was measured in the following reaction buffer: 45 mM EDTA, 30 mM sodium pyrophosphate, 1.425 M NaCl, 10 µM coelenterazine h (Promega) (Baker and Boyce, 2014).

Enzymatic activities were measured using a GloMax^®^-Multi Detection System (Promega) and the following program: 25 µl substrate; 2 s delay; 10 s measuring. Background luminescence was subtracted from each value and the results were normalized towards cells transfected with control siRNA.

### RNA Isolation and RT-qPCR and RNA Sequencing

The Power SYBR^®^ Green Cells-to-CT™” kit (Thermo Fisher Scientific) was used according to manufacturer’s protocol to isolate RNA, reverse transcribe it and synthesize cDNA. Quantitative PCR was performed in a CFX connect Thermocycler (Bio-Rad, Hercules, CA) using the following specific primers (Mauthe et al., 2016): HSBP1 (TATCGCGGACCTCATGACAC and TAGCAACCTTCAACTCTTTTGCG), ULK1 (TGGGCAAGTTCGAGTTCTCC and CTCCAAATCGTGCTTCTCGC), ULK2 (TGGAGACCTCGCAGATTATTTGC and ACACTCTGATCGTGTCTTCACT), ATG101 (TCCTCCAGCTTCCGAGTCCA and CCACGTAACCAGGGAGGAAC), FIP200 (CTCAAACCAGGTGAGGGTGCTTCA and TGTTTTGTGCCTTTTTGGCTTGACA), ATG13 (TCCAGACAGTTCGTGTTGGG and CTCAAATTGCCTGGTAGACATGA), GAPDH (GGGAACGCATTGACTGTTTT and CTCGGGCTTCTCAAAGTCAC).

The mRNA expression levels were first normalized against the expression of GAPDH, before comparing gene expression levels in HSPB1-depleted cells with those in the control cells.

### Statistical Analyses

Statistical significance was evaluated using two-tailed heteroscedastic *t*-testing before calculating the *p*-values. Individual data points from each independent experiment (the number of the independent experiments is indicated in each figure legend) were used to determine significances.

## Results

### HSBP1 Interacts With the ULK Kinase Complex Through FIP200

**Figure 1:**
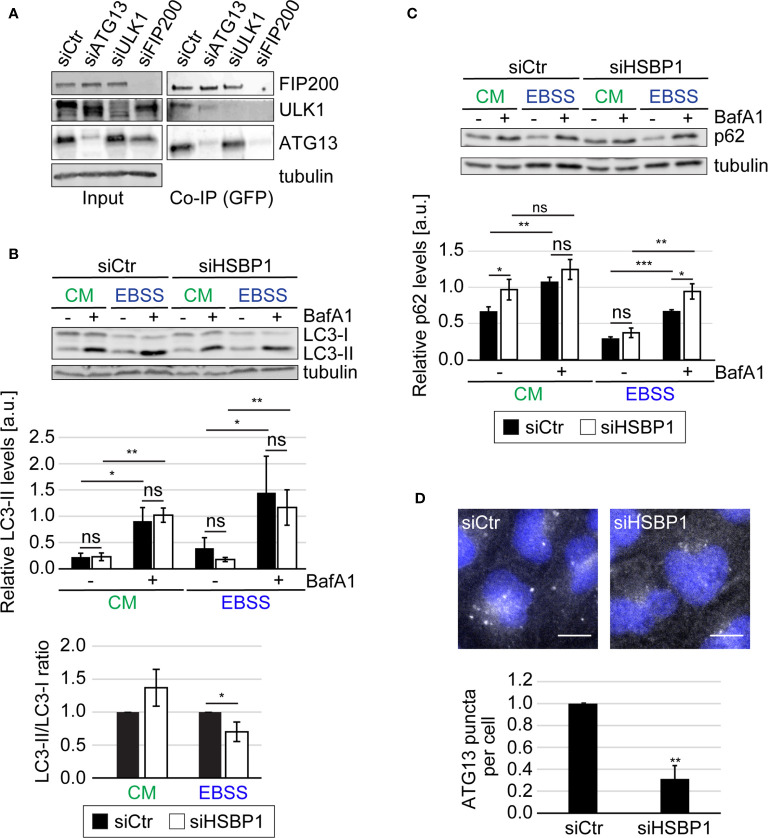
HSBP1 interacts with the ULK kinase complex. **(A)** GFP-HSBP1 U2OS cells were transfected with control siRNA (siCtr) or siRNAs against ATG13 (siATG13), ULK1 (siULK1) or FIP200 (siFIP200), lysed and subjected to co-immunoprecipitation using GFP‐trap beads 48 h after siRNA transfection. Input lysates and Co-IP were examined by WB using antibodies against ATG13, ULK1, FIP200 and tubulin. Tubulin served as the loading control. One representative blot is shown (n = 3). **(B, C)** U2OS cells were transfected with either siCtr or siHSBP1 for 48 h and then maintained in the control medium (CM) or transferred into EBSS to induce autophagy, in the presence (+) or the absence (-) of 200 nM BafA1 for 2 h. Cells were then lysed and proteins examined by WB using anti-LC3 **(B)**, anti-p62 **(C)** and anti-tubulin antibodies. Tubulin served as the loading control. LC3-II and p62 signals were normalized to tubulin (a.u., arbitrary units) and LC3-II/LC3-I ratios determined, and values are presented in the depicted graphs. Error bars represent the standard deviations (SDs) of 3 independent experiments. **(D)** U2OS cells were transfected with either siCtr or siHSBP1 for 48 h and then transferred into EBSS medium for 2 h, before being processed for IF using anti-ATG13 antibodies. Representative images are shown and the number of ATG13-positive puncta per cells was quantified. Error bars represent SDs of 3 independent experiments. Scale bars: 10 µm. The statistical significances were calculated to the controls. The symbols *, ** and *** indicate significant differences of p < 0.05, p < 0.01 and p < 0.001, respectively and ns indicate not significant.

We have found previously that FIP200 and ATG13 restrict picornavirus replication independently of their roles as components of the ULK kinase complex (Mauthe et al., 2016). We furthermore identified two potential binding partners that were shared by ATG13 and FIP200, i.e., HSBP1 and cell cycle progression 1 (CCPG1) (Mauthe et al., 2016). Since CCPG1 has recently been characterized as an ER-phagy receptor (Smith et al., 2018), we focused our attention to HSBP1 because no connection to autophagy or virus replication has been previously reported. To verify that HSBP1 is indeed interacting with FIP200 and ATG13, we performed Co-IP experiments in U2OS cells ectopically expressing GFP-HSBP1 (GFP-HSBP1 U2OS cells) using the GFP-Trap^®^ resin (**Figure 1A** and **Figure S1A**). We could confirm that GFP-HSBP1 specifically interacts with all the subunits of the tested ULK kinase complex, i.e. FIP200, ATG13 and ULK1 (**Figure S1A**). Next, we explored how HSBP1 binds to the ULK kinase complex also with Co-IP experiments. When ATG13 was knocked down in GFP-HSBP1 U2OS cells, the interaction between HSBP1 and ULK1, but not with FIP200 was abolished (**Figure 1A**). ULK1 knockdown did not influence the interaction of both FIP200 and ATG13 with HSBP1. In contrast, FIP200 depletion eliminated the binding between HSBP1 and ULK1 or ATG13 (**Figure 1A**). Altogether, these Co-IP experiments revealed that HSBP1 binds to the ULK kinase complex *via* FIP200.

### HSBP1 Is Required for Full Autophagy Induction

**Figure 2:**
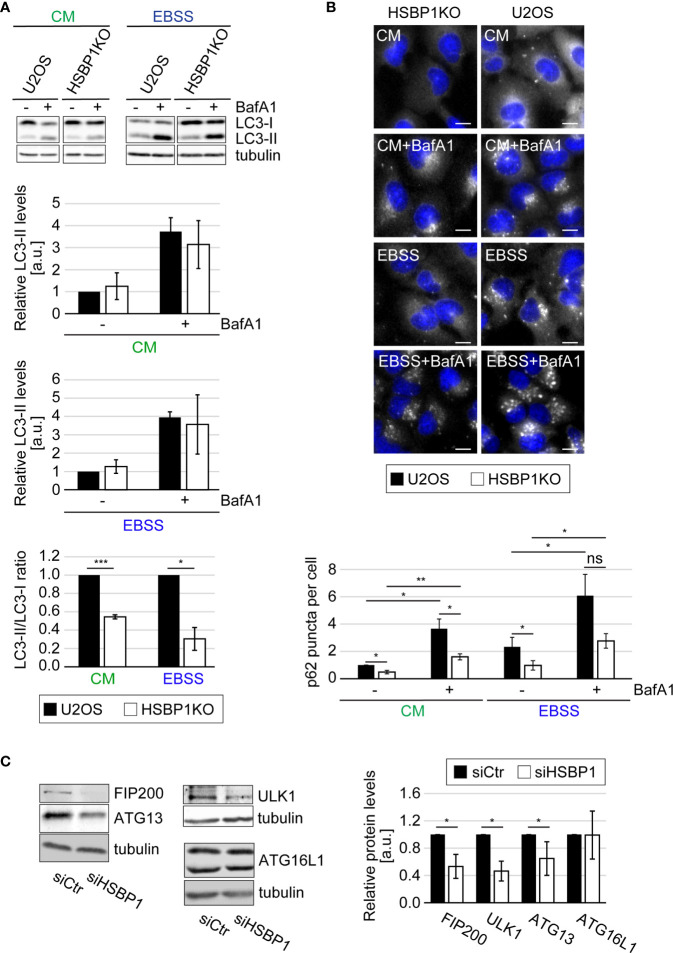
HSBP1 is required for full autophagy induction. **(A)** U2OS and HSBP1KO cells were maintained in CM or transferred into EBSS medium in the presence (+) or the absence (-) of 200 nM BafA1 for 2 h. Cells were subsequently lysed and WB performed using anti-LC3 and anti-tubulin antibodies. Tubulin served as the loading control. LC3-II WB signals were normalized to tubulin (a.u.) and LC3-II/LC3-I ratios determined. Error bars represent SDs of 3 independent experiments. **(B)** U2OS and HSBP1KO cells were kept in CM or transferred into EBSS medium in the presence (+) or the absence (-) of 200 nM BafA1 for 2 h. Cells were processed for IF using anti-p62 antibodies. Representative images are shown and the number of p62-positive puncta per cells was quantified. Scale bars: 10 µm. Error bars represent SDs of 3 independent experiments. **(C)** U2OS cells were transfected with either siCtr or siHSBP1 for 48 h before to be lysed and carrying WB analyses with antibodies against HSBP1, ATG13, FIP200, ULK1, ATG16L1 and tubulin. Tubulin was used as the loading control. Signal intensities were normalized to tubulin (a.u.). Error bars represent SDs of 3 independent experiments. The symbols *, ** and *** indicate significant differences of p < 0.05, p < 0.01 and p < 0.001, respectively and ns indicate not significant.

The ULK kinase complex is essential for autophagy initiation (Licheva et al., 2021). Therefore we tested whether HSBP1 depletion alters the autophagic response to nutrient deprivation (**Figures 1**, **2** and **Figures S1**, **S2**). First, we performed a classical autophagic flux assay (Klionsky et al., 2021) in which we treated control and HSBP1-depleted cells (**Figures S1B**, **S4**) with EBSS for 2 h, to induce autophagy in the presence or absence of BafA1, a lysosomal inhibitor (**Figures 1B, C**). As a readout, we measured the conversion of non-lipidated microtubule associated protein 1 light chain 3 (LC3/MAP1LC3)-I into lipidated, autophagosomal membrane-associated LC3-II (**Figure 1B**) and the levels of sequestosome 1 (p62/SQSTM1) (**Figure 1C**) by WB, and examined the RFP-GFP-LC3 fluorescent reporter by fluorescence microscopy (**Figure S1C**), which are all assays that allow to assess autophagy induction and progression (Klionsky et al., 2021). The WB analyses revealed that BafA1 treatment increased the LC3-II and p62 levels in control and HSBP1-depleted cells to a similar extend (**Figures 1B, C**). Moreover, the number of formed autolysosmes was also equal in these cells (**Figure S1C**). Together, these data clearly show that autophagic progression is not blocked in the absence of HSBP1. Nonetheless, we detected a reduced autophagosome formation rate under starvation conditions, i.e., lower ratio of LC3-II/LC3-I, in HSBP1-depleted cells in comparison to the control (**Figure 1B**). To confirm the reduced autophagosome formation rate, we treated control and HSBP1-depleted cells with EBSS for 2 h and quantified endogenous ATG13 puncta by immunofluorescence microscopy (IF), which represent autophagosome formation sites (Karanasios et al., 2013). Knockdown of HSBP1 caused a significant reduction in the number of ATG13 puncta per cell (**Figure 1D**). We also generated a HSBP1 knockout cell line in U2OS cells, HSBP1KO (**Figures S2A**, **S4**), and repeated the autophagic flux assay to corroborate the effects that HSBP1 depletion had on autophagy (**Figures 2A, B** and **Figure S2B**). Analogously to the result observed in HSBP1-depleted cells, we detected an impairment in autophagosome formation reflected by a lower LC3-II/LC3-I ratio (**Figure 2A**). We furthermore observed a significantly reduced number of p62 puncta per cell in HSBP1KO under control and starvation conditions (**Figure 2B**), whereas LC3 puncta were reduced under some conditions, but not significantly (**Figure S2B**). p62 forms discrete puncta when autophagy is induced and therefore measuring the amount of those is widely used as a read-out for autophagy induction (Orhon and Reggiori, 2017). Altogether, these results show that depletion of HSBP1, a novel interactor of the ULK kinase complex, impairs nutrient starvation-induced early autophagy events.

Two scenario could explain the negative impact that HSBP1 knockdown has on the nutrient-induced autophagic response. The first is that HSBP1 is a positive regulator for the ULK kinase complex. The second is that HSBP1 could be important to stabilize this complex, a possibility evoked by the fact that HSBP1 has been shown to be crucial in the assembly and stabilization of the WASH complex (Visweshwaran et al., 2018). This second scenario was tested by knocking down HSBP1 and examine the levels of the ULK kinase complex subunits by WB. Indeed, HSBP1 depletion led to a significant decrease of ATG13, FIP200 and ULK1 levels (**Figure 2C**). This effect was specific to these proteins since the expression levels of other ATG proteins, i.e. ATG16L1, was not influenced by HSBP1 knockdown. Since the decrease in protein expression was not due to a reduced mRNA expression (**Figure S2D**), this result shows that HSBP1 is important to stabilize the ULK kinase complex.

### HSBP1 Is a Novel Host Factor That Promotes EMCV and CVB3 Replication

**Figure 3:**
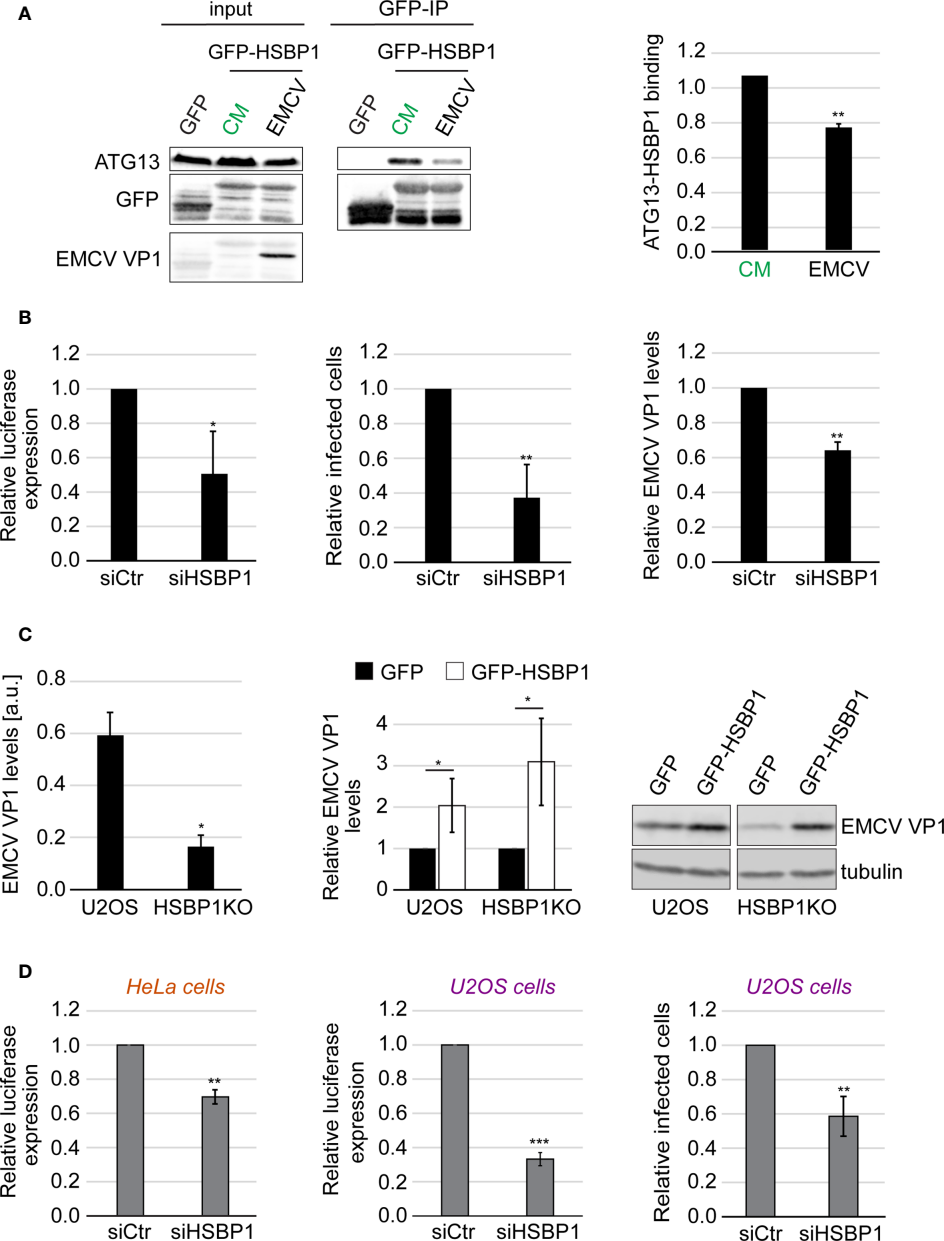
HSBP1 promotes EMCV and CVB3 replication. **(A)** U2OS cells were transfected with a plasmid carrying GFP or GFP-HSBP1 for 24 h and then kept in CM or were infected with EMCV for 6 h. Cells were then lysed and immunoprecipitated using GFP‐trap beads. Input lysates and Co-IP were examined by WB using antibodies against ATG13, GFP and EMCV VP1. Signal intensities were quantified and the of ATG13/GFP-HSBP1 ratios in the Co-IP were determined before to be normalized to that of the CM samples. Error bars represent SDs of 4 independent experiments. **(B)** U2OS cells were transfected with either siCtr or siHSBP1 for 48 h before being infected with EMCV or a luciferase-expressing EMCV strain (left panel) for 6 h. EMCV replication was quantified by luciferase expression (left panel), EMCV VP1-positive cells by IF (middle panel) and EMCV VP1 levels by WB (right panel). Error bars represent SDs of 3 (WB, right panel) or 4 independent experiments (luciferase and IF, left and middle panels). **(C)** U2OS and HSBP1KO cells were transfected with a plasmid expressing either GFP-HSBP1 or GFP for 24 h before being infected with EMCV for 6 h. Cells were then lysed and WB membranes probed with anti-EMCV VP1 and anti-tubulin antibodies. Tubulin was used as the loading control. Signal intensities were quantified and normalized to tubulin. EMCV VP1 levels in U2OS and HSBP1KO cells expressing GFP are compared in the left panel. EMCV VP1 levels in U2OS or HSBP1KO cells carrying GFP-HSBP1 were expressed relative to those in the same cells but carrying GFP (right panel). Error bars represent SDs of 4 independent experiments. **(D)** U2OS or HeLa cells were transfected with either siCtr or siHSBP1 for 48 h, before being infected with CVB3 (right panel) or a luciferase-expressing CVB3 strain (left and middle panels) for 6 h. CVB3 replication in HeLa cells was measured by luciferase expression (left panel) and in U2OS cells by either assessing luciferase expression (middle panel) or the percentage of CVB3 VP1-positive cells (right panel). Error bars represent SDs of 3 (HeLa cells) or 4 (U2OS cells) independent experiments. The statistical significances were calculated to the controls. The symbols *, ** and *** indicate significant differences of p < 0.05, p < 0.01 and p < 0.001, respectively.

Since HSBP1 binds to FIP200, we next examined whether HSBP1 also functions together with the ATG13-FIP200 subcomplex in controlling picornavirus infection. We repeated the Co-IP experiments using GFP-HSPB1 as a bait and measured the binding to ATG13 after either autophagy induction or EMCV infection (**Figure 3A**). EMCV is a member of the picornavirus family that we have previously employed to characterize the anti-picornaviral role of the FIP200-ATG13 subcomplex (Mauthe et al., 2016). The binding of HSBP1 to ATG13 remained unchanged upon autophagy induction (**Figure S3A**) showing that nutrient starvation and subsequent activation of the ULK kinase complex activity is not regulated through dynamic HSBP1 binding. In contrast, when the cells were exposed to EMCV, HSBP1 association with ATG13 was significantly reduced (**Figure 3A**). This result and the fact that HSBP1 binds the ULK complex *via* FIP200 (**Figure 1A**), suggests that HSBP1 is also involved and possibly regulates the ATG13-FIP200 subcomplex function in controlling the replication of EMCV and by extension, of other picornaviruses. To test whether HSBP1 influences EMCV replication, we infected control and HSBP1-depleted U2OS cells with a wild-type EMCV strain and quantified both viral capsid expression and the percentage of virus infected cells. In parallel, the same cells were also infected with a luciferase-expressing EMCV strain to assess virus replication by measuring luciferase activity (Mauthe et al., 2016). We found that HSBP1 knockdown reduced EMCV replication to 40-60% in comparison to control cells (**Figure 3B**). We also infected the HSBP1KO cells and observed that EMCV replication was significantly reduced, confirming the results obtained with the siRNA (**Figure 3C**). Conversely, GFP-HSBP1 overexpression led to an increase in EMCV replication, revealing that HSBP1 is a novel host factor that positively regulates EMCV propagation (**Figure 3C**). Finally, we also tested CVB3 replication in HSBP1-depleted cells to determine whether what we observed is EMCV specific or not. Measurement of luciferase expression and quantification of virus-positive cells in HeLa and U2OS cells upon HSBP1 knockdown (**Figure 3D**) and in HSBP1KO cells (**Figure S3B**) revealed HSBP1 depletion also reduced CVB3 replication. These data established HSBP1 as a novel host factor that is required for optimal EMCV and CVB3 replication.

### EMCV Infection Triggers GFP-HSBP1 Translocation From Cytoplasm Into the Nucleus

**Figure 4:**
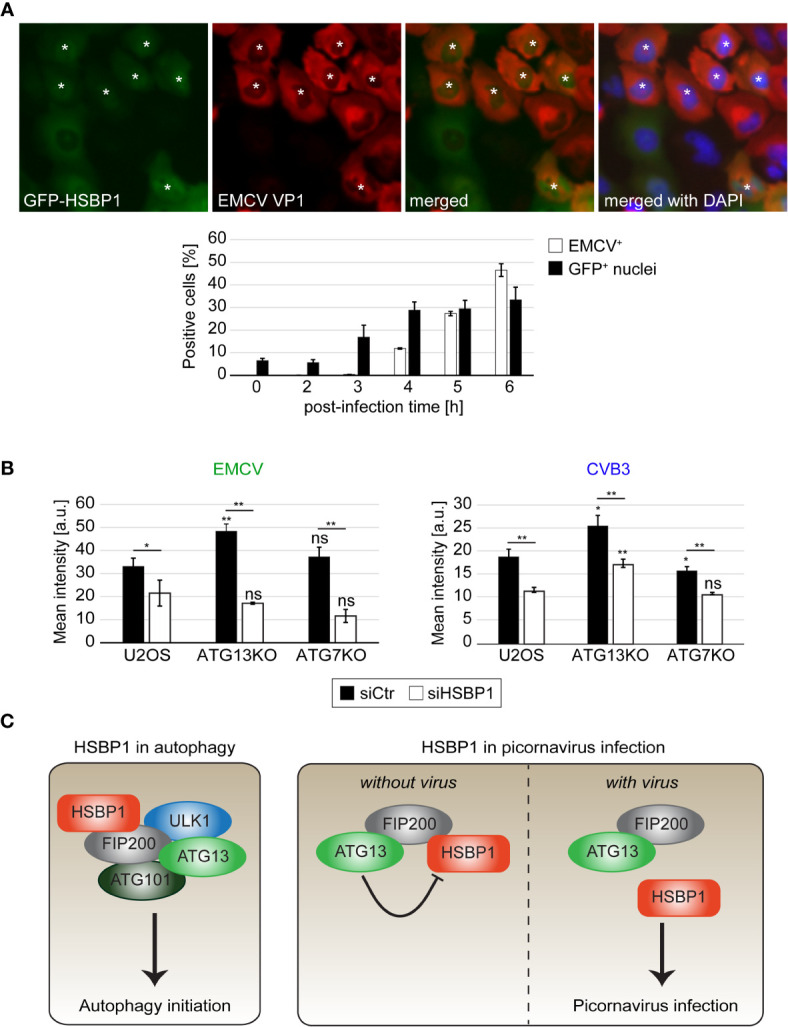
HSBP1 counteracts the anti-picornaviral function of ATG13 and translocates to the nucleus upon infection. **(A)** GFP-HSBP1 U2OS cells were infected with EMCV for the indicated times before being fixed and immunostained with anti-EMCV VP1 antibodies. IF images were automatically acquired and analyzed using the TissueFAXS microscope and software. EMCV VP1 positive cells (EMCV^+^) and cells positive for GFP-HSBP1 signal in the nucleus (GFP^+^ nuclei) were quantified. Error bars represent SDs of 3 independent experiments. White asterisks highlight GFP-HSBP1 U2OS cells with signal in the nucleus. **(B)** U2OS, ATG13KO and ATG7KO cells were transfected with either siCtr or siHSBP1 for 48 h, before being infected with EMCV (left panel) or CVB3 (right panel) for 6 h. Cells were immunostained using antibodies against EMCV and CVB3 VP1 to identify the infected cells and determine the degree of infection. The mean signal intensities were quantified and error bars represent SDs of 3 independent experiments. **(C)** Schematic model for the functions of HSBP1 in autophagy and picornavirus replication. The statistical significances were calculated to the controls. The symbols * and ** indicate significant differences of p < 0.05 and p < 0.01, respectively and ns indicate not significant. If not indicated otherwise, statistical differences were calculated to the control (U2OS cells).

Picornaviruses cause a so-called nucleocytoplasmic traffic disorder by modulating the nuclear pore complexes and thereby disrupting the regulated transport of material, i.e., proteins and mRNA, in and out of the nucleus (Lidsky et al., 2006; Lizcano-Perret and Michiels, 2021). Interestingly, we also observed a relocalization of GFP-HSBP1 from the cytoplasm into the nucleus upon EMCV infection by fluorescence microscopy (**Figure 4A**). This redistribution was already seen at 3 h post-infection, in agreement with the notion that the nucleocytoplasmic traffic disorder is an early event during EMCV infection (Lidsky et al., 2006), while the capsid protein expression was only detected at 4-6 h post-infection when more than 90% of the infected cells showed nuclear GFP-HSBP1 (**Figures S3C, D**). GFP-HSBP1 relocalization was strongly reduced when we inhibited virus replication by either treating cells with cycloheximide 1 h after exposure to EMCV or inoculating cells with inactivated EMCV, showing that HSBP1 redistribution is induced by an active EMCV infection (data not shown). Previous studies have demonstrated that EMCV-induced nucleocytoplasmic traffic disorder is caused by the viral Leader (L) protein, which alters the phosphorylation status and thereby the function of the nuclear pore complexes (Lidsky et al., 2006). We also found that GFP-HSBP1 translocation into the nucleus depends on L since infection with EMCV-Zn, an EMCV strain lacking L (Hato et al., 2007), did not lead to the same change (**Figures S3C, D**). The mechanism underlying nucleocytoplasmic traffic disorders differs between members of the cardioviruses (*Picornaviridae*) like EMCV and members of the enteroviruses (*Picornaviridae*) such as CVB3 and EV71 (Lizcano-Perret and Michiels, 2021). Thus, we tested whether the relocalization of HSBP1 is specific to EMCV or also occurs upon infection with CVB3 and EV71. To this aim, we infected GFP-HSBP1 U2OS cells with EV71 and CVB3 before imaging them. Cell infection with these viruses also caused a relocalization of HSBP1 from the cytoplasm into the nucleus, i.e. more than 90% of infected cells displayed nuclear GFP-HSBP1 (**Figures S2**, **3C, D**), showing that HSBP1 redistribution is triggered by all the tested picornaviruses. Although it remains unclear whether the presence of HSBP1 in the nucleus is beneficial for the virus or simply a consequence of the nucleocytoplasmic traffic disorder, it is clearly specific for picornaviruses since viruses from other families (e.g., IAV, ZIKV, DENV or CHIKV) or treatments triggering autophagy *via* endoplasmic reticulum stress, did not cause HSBP1 translocation into the nucleus (**Figures S3E, F**, and data not shown).

### The Anti-Picornaviral Effect of the FIP200-ATG13 Subcomplex Involves HSBP1

Replication of EMCV and other picornaviruses is enhanced when ATG13 and FIP200 are depleted (Mauthe et al., 2016). Since HSBP1 depletion has an opposite effect, we examined the functional relationship between these three proteins. To this aim, we infected wild type, ATG7KO (Janssen et al., 2018) and ATG13KO (**Figure S2C**) cells after knocking down or not HSBP1, and then measured EMCV infection by IF (**Figure 4B**). In agreement with previous results (Mauthe et al., 2016), EMCV replication was more efficient in the ATG13KO than in wild type and ATG7KO cells. Interestingly, HSBP1 depletion reduced EMCV infection also in ATG13KO cells, when compared to control siRNA treated cells (**Figure 4B**). To confirm that this observation is not specific for EMCV infection, we repeated the same experiment using CVB3, obtaining an identical result (**Figure 4B**). Collectively, these data show that the effect of HSBP1 on picornavirus replication is not mediated through its function in autophagy since HSBP1 depletion decreased viral replication in ATG7KO cells. Moreover, these results indicate that the anti-picornaviral function of the FIP200-ATG13 subcomplex is connected to HSBP1, since after the depletion of HSBP1 in ATG13KO cells (**Figure 4B**), the effect caused by the loss of ATG13 and FIP200 is lost.

## Discussion

Autophagy is an important cellular survival mechanism that keeps cellular homeostasis under several stress conditions (Lahiri et al., 2019). The ULK kinase complex is crucial for autophagy initiation, and its function is modulated through the interaction of its subunits with various binding partners, including SMCR8-C9orf72 complex subunit (SMCR8) (Yang et al., 2016), autophagy and beclin 1 regulator 1 (AMBRA1) (Nazio et al., 2013) and acidic lipids (Karanasios et al., 2013). Here, we identified a novel ULK kinase complex binding partner, HSBP1, which interacts with this complex *via* FIP200 (**Figure 4C**). We have found that HSBP1 knockdown decreases the formation rate of autophagosomes but does not influence the overall autophagic flux. Since the depletion of HSBP1 caused a reduction in the levels of ULK kinase complex components, we suggest that HSBP1 could be involved in the assembly and/or stability of this complex in a similar manner as it has been shown for the WASH complex (Visweshwaran et al., 2018). However, the autophagy defect caused by HSBP1 depletion is much less severe than when an ULK kinase complex component is ablated, e.g. ATG13, since this causes a severe autophagic flux impairment (**Figure S2C**). Thus, HSBP1 has more a regulatory role and it might be required to fine tune the activity of this complex under specific conditions. Alternatively, HSBP1 may act as a chaperone promoting the assembly of the ULK kinase complex.

We have also found that HSBP1 knockdown reduces picornavirus replication, establishing HSBP1 as a novel host cell regulator of picornavirus infection. It has been shown that autophagy induction promotes picornavirus replication under some circumstances (Klein and Jackson, 2011; Huang and Yue, 2020), which could explain why HSBP1 depletion has a negative impact on picornavirus propagation. However, we detected a negative effect of HSBP1 depletion on EMCV replication also in autophagy deficient cells (**Figure 4B**). This result shows that the proviral function of HSBP1 is autophagy independent. In fact, we found that HSBP1 suppresses the anti-viral function of the FIP200-ATG13 subcomplex that we have previously discovered (**Figure 4C**). Thus, our data favors a possible model in which through binding HSBP1, the FIP200-ATG13 subcomplex inhibits the pro-picornaviral function of HSBP1. Upon picornaviral infection, HSBP1 dissociates from this complex and is released from the inhibitory action of FIP200-ATG13 subcomplex, fulfilling its proviral function. This would mean that the FIP200-ATG13 subcomplex carries out its anti-picornaviral function by preventing the pro-picornaviral function of HSBP1. This notion is corroborated by the observation that both the anti-picornaviral role of FIP200-ATG13 subcomplex is abolished when HSBP1 is depleted (**Figure 4B**) and picornavirus replication is reduced under the same situation (**Figure 3**). This notion is further supported by the finding that HSBP1 overproduction enhances EMCV replication (**Figure 3C**), possibly because the levels of FIP200-ATG13 subcomplex are insufficient to inhibit excess HSBP1. In line with this consideration, FIP200 overexpression reduces picornavirus replication (Mauthe et al., 2016), probably because the higher FIP200 levels can more effectively inhibiting HSBP1. Although this model can explain our data, we cannot exclude that HSBP1 functions in a step of the picornaviral life cycle that is epistatic to the one controlled by the FIP200-ATG13 subcomplex. For example, the disruption of the WASH complex by HSBP1 depletion (Visweshwaran et al., 2018) could negatively affect picornaviral cell entry. Consequently, HSBP1 could have even a dual role in this scenario, i.e., promoting cell entry through the stabilization of the WASH complex (or other yet uncharacterized functions of HSBP1) and counteracting the virus by stabilizing the FIP200-ATG13 subcomplex.

The nuclear relocalization of HSBP1 observed in picornavirus-infected cells is not associated with a defect in the general protein shuttling between the cytoplasm and the nucleus because viruses such as DENV and ZIKV, which are known to target nuclear pore complexes (Wubben et al., 2020), did not trigger HSBP1 redistribution into the nucleus (**Figures S3E, F**). An early report identified HSBP1 as a negative regulator of HSF1 and showed that it inhibits HSF1 function in the nucleus (Satyal et al., 1998). However, we have no indications of a possible functional connection between enhanced HSF1 activity and HSBP1 relocalization, since HSBP1 did not move to the nucleus upon ER stress caused by tunicamycin (data not shown), which is known to induce HSF1 activity (Liu and Chang, 2008). Additionally, HSF1 knockdown does not interfere with HSBP1 subcellular distribution (data not shown). Since HSBP1 has no apparent nuclear localization signals, we cannot exclude that the translocation into the nucleus is facilitated by a yet unknown binding partner. It also still remains to be determined whether HSBP1 fulfills its pro-picornaviral function within the nucleus or not.

In conclusion, we identified two new functions of HSBP1, both mediated *via* its binding to FIP200. On the one hand, HSBP1 regulates the early steps of autophagy by stabilizing the ULK kinase complex, and on the other hand, it functions as a positive regulator for picornavirus replication independently of its function in autophagy. Future studies will be required to unveil how HSBP1 is exactly promoting in the picornavirus life cycle.

## Data Availability Statement

The original contributions presented in the study are included in the article/**Supplementary Material**. Further inquiries can be directed to the corresponding author.

## Author Contributions

MM and FR designed the study. MM, NB, PV, and ND performed experiments. MM and FR wrote the manuscript. All authors contributed to the article and approved the submitted version.

## Funding

FR is supported by ZonMW TOP (91217002), Open Competition ENW-KLEIN (OCENW.KLEIN.118), and Marie Skłodowska Curie ETN (765912) grants. FR and ND are also supported by a Marie Skłodowska-Curie Cofund grant under the European Union’s Horizon 2020 Research and Innovation Programme PRONKJEWAIL (Grant Agreement No 713660).

## Conflict of Interest

The authors declare that the research was conducted in the absence of any commercial or financial relationships that could be construed as a potential conflict of interest.

## Publisher’s Note

All claims expressed in this article are solely those of the authors and do not necessarily represent those of their affiliated organizations, or those of the publisher, the editors and the reviewers. Any product that may be evaluated in this article, or claim that may be made by its manufacturer, is not guaranteed or endorsed by the publisher.

## Supplementary Material

Supplementary Figure 1 HSBP1 binds the ULK kinase complex components. **(A)** U2OS cells were transfected with plasmids expressing GFP-HSBP1 or GFP for 24 h. Cells were subsequently lysed and co-immunoprecipitated using GFP‐trap beads. Input lysates and Co-IP were examined by WB using antibodies against GFP, ATG13, ULK1 and FIP200. **(B)** HSBP1 levels in U2OS cells treated with siHSBP1 for 48 h, were assessed by WB. Tubulin is used as the loading control. **(C)** RFP-GFP-LC3 HeLa cells were transfected with either siCtr or siHSBP1 for 48 h and kept in CM or transferred into EBSS medium in the presence (+) or the absence (-) of 200 nM BafA1 for 2 h. Cells were fixed and images were automatically acquired and analysed. Representative images are shown and the number of autophagosomes (GFP-positive LC3 puncta) and autolysosomes (RFP-only positive LC3 puncta) per cell was quantified. Scale bars: 10 µm. Error bars represent SDs of 4 independent experiments. The symbols * and ** indicate significant differences of p < 0.05 and p < 0.01, respectively.

Supplementary Figure 2 HSBP1 depletion does not impair autophagy progression and ULK kinase complex component mRNA expression. **(A)** HSBP1 levels in HSBP1KO cells were assessed by WB. Tubulin is used as the loading control. **(B)** U2OS and HSBP1KO cells were kept in CM or transferred into EBSS medium in the presence (+) or the absence (-) of 200 nM BafA1 for 2 h. Cells were processed for IF using anti-LC3 antibodies. Representative images are shown and the number of LC3-positive puncta per cells was quantified. Scale bars: 10 µm. Error bars represent SDs of 3 independent experiments. **(C)** U2OS and ATG13KO cells were kept in CM or transferred into EBSS medium in the presence (+) or the absence (-) of 200 nM BafA1 for 2 h, before to be lysed and WB probed with antibodies recognizing ATG13, LC3 and actin. **(D)** U2OS cells were transfected with either siCtr or siHSBP1 for 48 h. Cells were subsequently lysed and mRNA levels of HSBP1, ATG13, FIP200 and ULK1 were measured using quantitative real-time PCR. Error bars represent SDs of 4 independent experiments. The symbol *** indicates a significant difference of p<0.001.

Supplementary Figure 3 Nuclear translocation of HSBP1 is specifically triggered by picornavirus infection. **(A)** U2OS cells were transfected with a plasmid carrying GFP-HSBP1 for 24 h and then kept in CM or transferred into EBSS medium for 2 h. Cells were then lysed and immunoprecipitated using GFP‐trap beads. Input lysates and Co-IP were examined by WB using antibodies against ATG13 and GFP. Signal intensities were quantified and the ATG13/GFP-HSBP1 ratios in the Co-IP were determined before to be normalized to that of the CM samples. Error bars represent SDs of 4 independent experiments. **(B)** CVB3 replication in HSBP1KO cells was measured by either assessing luciferase expression (left panel) or determining the percentage of CVB3 VP1-positive cells (right panel). Error bars represent SDs of 3 (left panel) or 6 (right panel) independent experiments. The statistical significances were calculated to the controls. **(C)** GFP-HSBP1 U2OS cells were infected with EMCV, EMCV-Zn, EV71 and CVB3 for 6 h before being fixed and immunostained with anti-EMCV VP1 (for EMCV and EMCV-Zn), anti-CVB3 VP1 (for CVB3) or anti-dsRNA (for EV71) antibodies. Images were automatically acquired and analyzed using the TissueFAXS microscope and software. The average percentage of virus positive cells +/- SD is indicated. **(D)** Virus positive cells with the GFP-HSBP1 signal in the nucleus (GFP+ nuclei) were quantified. Error bars represent SDs of 5 independent experiments. **(E)** GFP-HSBP1 U2OS cells were infected with EMCV (for 6 h), CHIKV (for 10 h), DENV (for 26 h), ZIKV (for 26 h) or IAV (for 6 h). Cells were then fixed and immunostained with virus specific antibodies before automatically acquire images and analyze them using the TissueFAXS microscope and software. The average percentage of virus positive cells +/- SD is indicated. **(F)** Virus positive cells with GFP-HSBP1 signal in the nucleus (GFP^+^ nuclei) were quantified. Error bars represent SDs of 3 or 4 independent experiments. The symbols ** and *** indicate significant differences of p < 0.01 and p < 0.001, respectively.

Supplementary Figure 4 Depletion of HSBP1 in U2OS cells. **(A)** Original, uncropped WB used for **Figure S1B**. HSBP1 and tubulin bands used for figure S1B are indicated in red squares. **(B)** Original, uncropped WB used for **Figure S2A**. HSBP1 and tubulin bands used for **Figure S2A** are indicated in red squares.
