# Supplementary figures and images for: HSBP1 Is a Novel Interactor of FIP200 and ATG13 That Promotes Autophagy Initiation and Picornavirus Replication
Source: Front Cell Infect Microbiol. 2021 Nov 15;11:745640. doi: 10.3389/fcimb.2021.745640 (PMC8634480; doi:10.3389/fcimb.2021.745640)

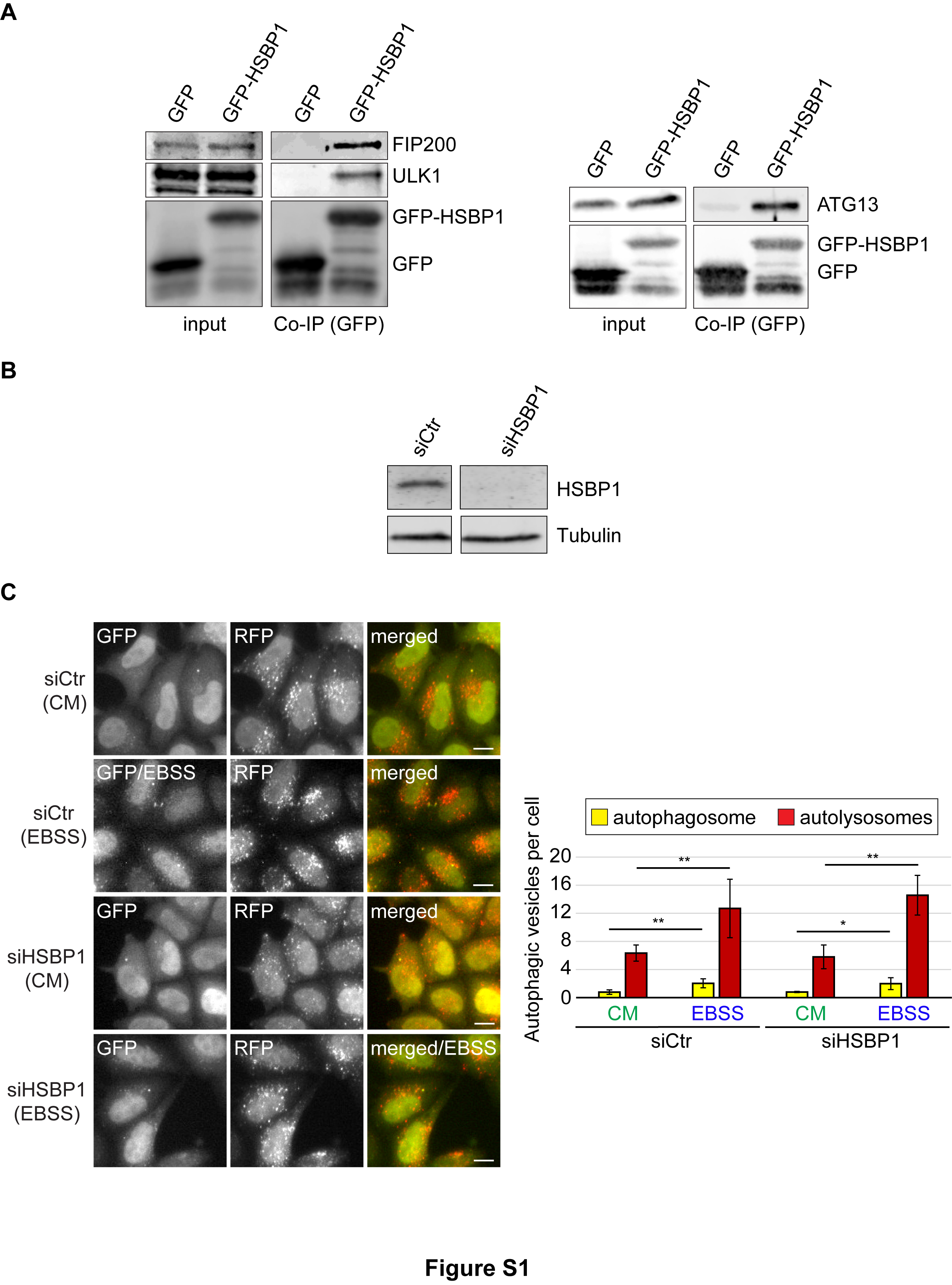

Supplement: Supplementary Figure 1 — HSBP1 binds the ULK kinase complex components. (A) U2OS cells were transfected with plasmids expressing GFP-HSBP1 or GFP for 24 h. Cells were subsequently lysed and co-immunoprecipitated using GFP‐trap beads. Input lysates and Co-IP were examined by WB using antibodies against GFP, ATG13, ULK1 and FIP200. (B) HSBP1 levels in U2OS cells treated with siHSBP1 for 48 h, were assessed by WB. Tubulin is used as the loading control. (C) RFP-GFP-LC3 HeLa cells were transfected with either siCtr or siHSBP1 for 48 h and kept in CM or transferred into EBSS medium in the presence (+) or the absence (-) of 200 nM BafA1 for 2 h. Cells were fixed and images were automatically acquired and analysed. Representative images are shown and the number of autophagosomes (GFP-positive LC3 puncta) and autolysosomes (RFP-only positive LC3 puncta) per cell was quantified. Scale bars: 10 µm. Error bars represent SDs of 4 independent experiments. The symbols * and ** indicate significant differences of p < 0.05 and p < 0.01, respectively. [file Image_1.tif]

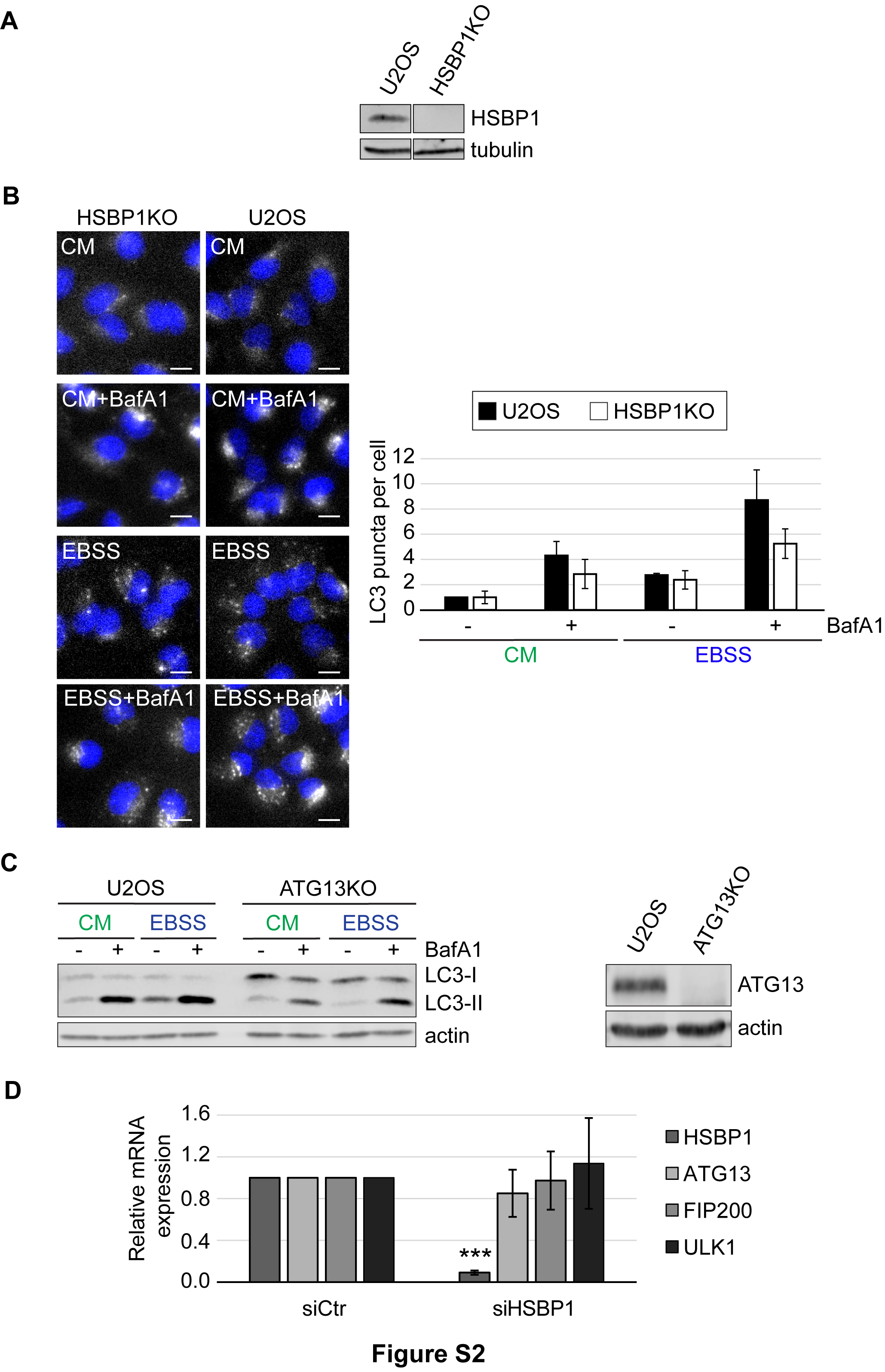

Supplement: Supplementary Figure 2 — HSBP1 depletion does not impair autophagy progression and ULK kinase complex component mRNA expression. (A) HSBP1 levels in HSBP1KO cells were assessed by WB. Tubulin is used as the loading control. (B) U2OS and HSBP1KO cells were kept in CM or transferred into EBSS medium in the presence (+) or the absence (-) of 200 nM BafA1 for 2 h. Cells were processed for IF using anti-LC3 antibodies. Representative images are shown and the number of LC3-positive puncta per cells was quantified. Scale bars: 10 µm. Error bars represent SDs of 3 independent experiments. (C) U2OS and ATG13KO cells were kept in CM or transferred into EBSS medium in the presence (+) or the absence (-) of 200 nM BafA1 for 2 h, before to be lysed and WB probed with antibodies recognizing ATG13, LC3 and actin. (D) U2OS cells were transfected with either siCtr or siHSBP1 for 48 h. Cells were subsequently lysed and mRNA levels of HSBP1, ATG13, FIP200 and ULK1 were measured using quantitative real-time PCR. Error bars represent SDs of 4 independent experiments. The symbol *** indicates a significant difference of p<0.001. [file Image_2.tif]

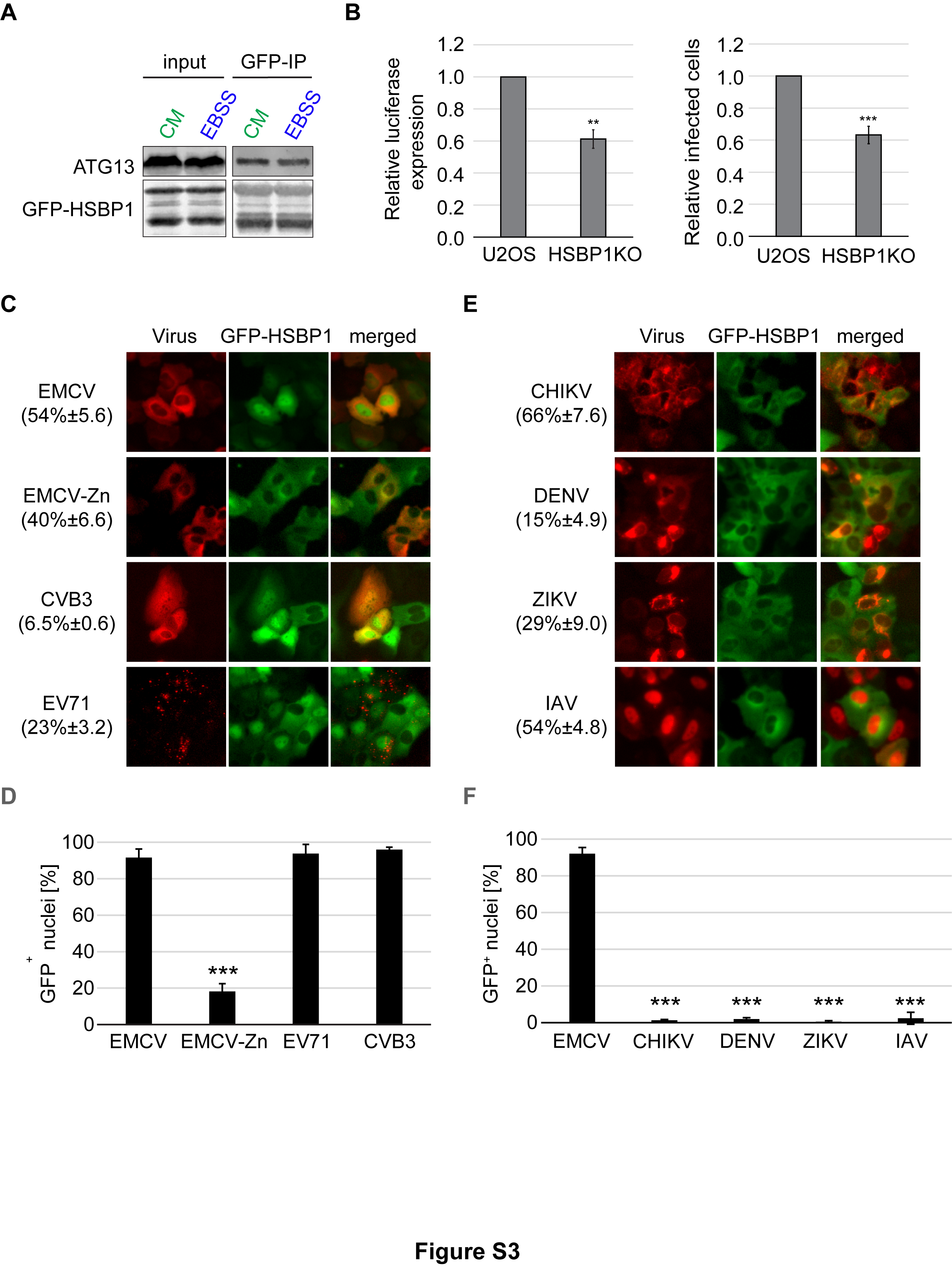

Supplement: Supplementary Figure 3 — Nuclear translocation of HSBP1 is specifically triggered by picornavirus infection. (A) U2OS cells were transfected with a plasmid carrying GFP-HSBP1 for 24 h and then kept in CM or transferred into EBSS medium for 2 h. Cells were then lysed and immunoprecipitated using GFP‐trap beads. Input lysates and Co-IP were examined by WB using antibodies against ATG13 and GFP. Signal intensities were quantified and the ATG13/GFP-HSBP1 ratios in the Co-IP were determined before to be normalized to that of the CM samples. Error bars represent SDs of 4 independent experiments. (B) CVB3 replication in HSBP1KO cells was measured by either assessing luciferase expression (left panel) or determining the percentage of CVB3 VP1-positive cells (right panel). Error bars represent SDs of 3 (left panel) or 6 (right panel) independent experiments. The statistical significances were calculated to the controls. (C) GFP-HSBP1 U2OS cells were infected with EMCV, EMCV-Zn, EV71 and CVB3 for 6 h before being fixed and immunostained with anti-EMCV VP1 (for EMCV and EMCV-Zn), anti-CVB3 VP1 (for CVB3) or anti-dsRNA (for EV71) antibodies. Images were automatically acquired and analyzed using the TissueFAXS microscope and software. The average percentage of virus positive cells +/- SD is indicated. (D) Virus positive cells with the GFP-HSBP1 signal in the nucleus (GFP+ nuclei) were quantified. Error bars represent SDs of 5 independent experiments. (E) GFP-HSBP1 U2OS cells were infected with EMCV (for 6 h), CHIKV (for 10 h), DENV (for 26 h), ZIKV (for 26 h) or IAV (for 6 h). Cells were then fixed and immunostained with virus specific antibodies before automatically acquire images and analyze them using the TissueFAXS microscope and software. The average percentage of virus positive cells +/- SD is indicated. (F) Virus positive cells with GFP-HSBP1 signal in the nucleus (GFP+ nuclei) were quantified. Error bars represent SDs of 3 or 4 independent experiments. The symbols ** and *** indicate significant [file Image_3.tif]

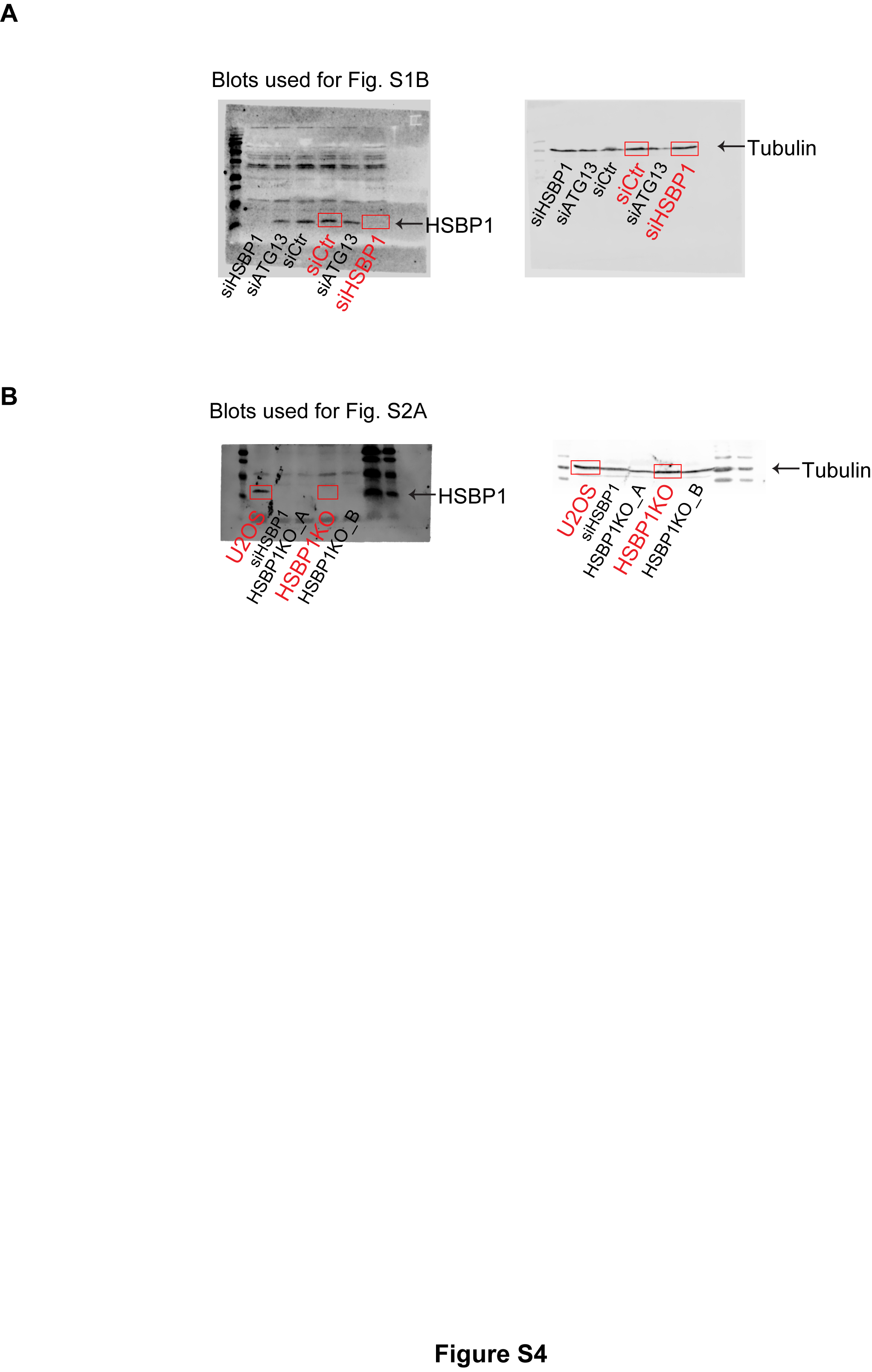

Supplement: Supplementary Figure 4 — Depletion of HSBP1 in U2OS cells. (A) Original, uncropped WB used for Figure S1B . HSBP1 and tubulin bands used for figure S1B are indicated in red squares. (B) Original, uncropped WB used for Figure S2A . HSBP1 and tubulin bands used for Figure S2A are indicated in red squares. [file Image_4.tif]
